# Developing and Validating the Teacher Rating Scale of Learning Interest for Kindergarteners

**DOI:** 10.3389/fpsyg.2022.890328

**Published:** 2022-05-19

**Authors:** Chung Chin Wu

**Affiliations:** Department of Early Childhood Education, National Pingtung University, Pingtung, Taiwan

**Keywords:** individual interest, kindergarteners, learning interest, measurement invariance, situational interest

## Abstract

Studies have investigated learning interest based on either 2- or 4-factor theoretical framework. Empirical studies showed supportive evidence only toward the 2-factor learning interest model, but it was primarily demonstrated above the secondary level. It is unclear whether the dimensionality of the learning interest of kindergarteners is consistent with those studies conducted above the secondary level due to the absence of an instrument for measuring kindergarteners' learning interests. An effective and efficient learning interest scale was developed and validated for teachers' use to rate kindergarteners to provide useful information for improving teaching and learning in practice. A total of 132 5-year-old kindergarteners were rated by 5 teachers in the formal study. The results clearly showed: (1) the developed teacher rating scale of learning interest was valid for understanding kindergarteners' learning interests and was equally suitable for boys and girls. (2) The 2-factor learning interest model was the best theoretical viewpoint for understanding kindergarteners' learning interests across gender. The implications for learning interest research and practice are also discussed.

## Introduction

The learning interest construct has been proposed over three decades (Hidi, 1990). To date, learning interest is still an ongoing discussed topic in academic and in practice (i.e., Nuutila et al., 2018; Xu, 2018; Murayama et al., 2019; Shum et al., 2020; Fryer et al., 2021). For educators, it was always one of the main concerns and the most challenging task about how to engage students in the learning process, especially those unmotivated students (Hidi and Harackiewicz, 2000). Teachers usually do not have a clear understanding of whether their instructional practice or which component of their instructional design can affect developing students' interests (Hidi and Renninger, 2006). It was especially the case for teachers in kindergartens because curriculum and learning activities were independently designed by teachers without referencing textbooks. Consequently, it may result in declined learning interest with age (Chittum et al., 2017). It is crucial to develop a learning interest scale for kindergarten teachers to recognize the effect of their instruction on motivating children, and it can also provide useful information for revising their instructional design and content in the future.

Learning interest reflects positive affect, cognition, and attractiveness when students are engaging in the learning process (Hidi et al., 2004). In the early stage, learning interest was operationalized into two dimensions: situational interest and individual interest. Individual interest reflected long-term and stable characteristics and it was relatively independent of context. Situational interest focused on how contextual factor affects the interest of most subjects (Hidi, 1990), and it may be triggered by interesting learning materials or activities, and it may, in turn, contribute to raising positive eeffect (i.e., feel interesting) in the class (Mitchell, 1993; Schraw et al., 2001; Linnenbrink-Garcia et al., 2010; Rotgans and Schmidt, 2011). Situational interest has the potential to turn into individual interest if it can be maintained in the learning process for a period (Hidi, 1990; Harackiewicz et al., 2000; Ainley, 2006).

Hidi and Renninger (2006) further proposed four development stages based on the 2-factor framework of learning interest, in which situational interest was decomposed into triggered situational interest and maintained situational interest, whereas individual interest was decomposed into emerging individual interest and well-developed individual interest. Triggered situational interest is posited to emerge when an interesting teaching method is used by a teacher, whereas maintained situational interest is observed when someone is experiencing enjoyment in the learning process. Emerging individual interest is documented when a student is observed to try solving challenging tasks, whereas well-developed individual interest is considered to develop when someone actively tries different ways to resolve difficult problems. These four stages of learning interest were considered to be developed in sequence from triggered situational interest to well-developed individual interest, and the later development stage emerged once the former stage was developed and maintained for a period (Krapp and Lewalter, 2001; Lipstein and Renninger, 2006). Altogether, it suggested that learning interest may be composed of either 2 or 4 factors. However, empirical studies only showed supportive evidence toward the 2-factor learning interest model.

A few researchers adopted either 1-factor (situational or individual interest) (Harackiewicz et al., 2000; Chittum et al., 2017) or 2-factor learning interest in the study without addressing on clarifying the factorial structure of learning interest (Hulleman et al., 2008). More recently, researchers conducted confirmatory factor analysis to investigate the factorial structure of learning interest in secondary school students, and found that situational interest may include the content of triggered and maintained situational interest whereas individual interest was just a single factor rather than 2 factors (emerging and well-developed individual interests) as it was proposed in the four-stage interest theory (Linnenbrink-Garcia et al., 2010). There was also some indirect evidence that showed supportive evidence toward the 2-factor model on secondary and college students (Harackiewicz et al., 2008; Knogler et al., 2015). It suggested that the 2-factor learning interest model is better than the 4-factor model for researchers or practitioners to understand students' learning interests.

To date, learning interests were only examined above the elementary school level (Schiefele et al., 1992; Harackiewicz and Elliot, 1993; Harackiewicz et al., 1997, 2000, 2002; Senko and Harackiewicz, 2005; Hulleman et al., 2008; Chittum et al., 2017; Friedman et al., 2017; Grigg et al., 2018; Nuutila et al., 2018). It was unclear whether kindergarteners' learning interest in language learning was developed at an early stage and the dimensionality of kindergarteners' learning interest was consistent with the former findings. whether the dimensionality of kindergarteners' learning interest was consistent with the former findings due to the absence of a valid instrument for measuring kindergarteners' learning interest. Consequently, the purposes of this study were two-fold:

(1) To develop an effective instrument for the teacher to measure kindergartener's learning interest.
(2) To examine the best theoretical perspective for understanding the learning interest of kindergarten boys and girls.

## Methodology

### Participants

A pilot study was carried out to assure that teachers were correctly understanding the wording in the instrument. A total of 4 kindergarten teachers rated a total of 41 kindergarteners in the pilot study. In the formal study, a total of 132 (68 males and 64 females) 5-year-old kindergarteners selected from five kindergarten classes were rated respectively by 5 teachers teaching in different kindergartens. The teachers were all females aged 26–46 years (*M* = 36.8, *SD* = 7.95), and they implemented a thematic teaching model which was one of the most common teaching models in practice in the early childhood education stage. The kindergarteners in these five classes received a similar main course arrangement with 1 h for outdoor activity, 1 h for free play in the learning area, and forty to one and half hour for thematic activity. The language course was designed to teach Hakka Chinese to kindergarteners to preserve the minor language. There was a full 40 min once a week for teachers to implement the language course. In this class, teachers designed related activities and used Hakka Chinese to teach kindergarteners to know the common pronunciation and simple conversation in daily life. In thematic learning activities, teachers used Chinese and Hakka Chinese alternately on occasions. The participants consented to participate in this study and were guaranteed that their responses would be kept confidential.

### Instruments

The teacher rating scale of learning interest for kindergarteners was developed based on the 2-factor learning interest to measure the kindergarteners' learning interest in language class (Hidi, 1990), and it was composed of two subscales, respectively, for measuring situational interest and individual interest. The subscale of situational interest included items for measuring triggered and maintained situational interest to better capture this construct which was suggested by empirical findings (Linnenbrink-Garcia et al., 2010). Originally, a total of 10 items were developed according to the learning interest theory. After an assessment by two raters, eight items which were considered the most relevant and fitted the definition of situational and individual interests were retained in the current instrument. Finally, a total of eight items were formed to measure the 2-factor learning interest. Each factor was measured with four items. It should be noted that the results of the pilot study did not suggest any required amendments in the wordings for this scale. Consequently, the scale used in the pilot study was adopted in the formal study. Description in each item was assigned a character role with the same pseudonyms, and this design was demonstrated to be an effective way for the teacher to rate a specific kindergartener (Wu, 2022). Four items measuring situational interest were as follows: (1) Bob is attracted by the teaching method and teaching material. (2) Bob enjoys coming to class. (3) Language classes in kindergarten fascinate Bob. (4) Bob feels that language classes are interesting. The former and the latter two items are respectively designed to capture triggered situational interest and maintained situational interest. Another four items measuring individual interest were as follows: (1) Bob enjoys learning tasks very much. (2) Bob tries to answer different questions using taught language. (3) Bob likes language classes more than other classes. (4) Bob devotes extra time to learning language after the language class. After completely reading each statement of the item, teachers were asked to select one from the six options on a 1 (“completely mismatch”) to 6 (“completely match”) scale, which denoted to what extent a child matches or mismatches to that statement based on teacher's observations and understandings of those children in learning activities.

### Analysis

Before conducting primary analysis, inter-rater reliability was calculated to assure the rating consistency between raters. The inter-rater reliability coefficient for eight items ranged from 0.85 to 0.92, which indicated a high rating consistency. To identify the best theoretical model for understanding kindergarteners' learning interests, three primary theoretical models, respectively, encompassing 1- factor, 2-factor, and 3-factor learning interests were set up and analyzed. The 1-factor model was constructed by rearranging the items developed based on the 2-factor model, and these items were loaded together on a single factor called learning interest. For the 2-factor learning interest model, each of the 4 items for measuring situational and individual interests was respectively loaded on their posited factors. For the 3-factor learning interest model, each of the 2 items for measuring situational interest was respectively loaded on triggered and maintained situational interest while the other four items were loaded together on a single factor (individual interest).

Confirmatory factor analyses (CFAs) were implemented, and χ^2^, CFI, TLI, and RMSEA were used to assess the goodness of fit of the three models to the data. CFI ≥ 0.95, TLI ≥ 0.95, and RMSEA ≤ 0.08 indicated that the model fitted the data well (Hu and Bentler, 1999; Wang and Wang, 2012). Δχ^2^, Akaike Information Criterion (AIC), and Bayesian Information Criterion (BIC) were used to compare the parsimoniousness of the models. Δχ^2^ was calculated by subtracting the χ^2^ value of the k+1 factor (i.e., 2-factor) model from the k factor (i.e., 1-factor) model. Significant Δχ^2^ value, lower AIC, and lower BIC values indicated that the k+1 factor model was the best theoretical model. In contrast, a non-significant Δχ^2^ value between the k factor (i.e., 2-factor) model and k+1 factor (i.e., 3-factor) model indicated the more complex (i.e., 3-factor) model does not contribute to the goodness of fit of that model to the data. If this is the case, the k factor (i.e., 2-factor) model was demonstrated to be the best theoretical model.

After identifying the best model, standardized factor loadings, individual item reliability, composite reliability (CR), and average variance extracted (AVE) were used to examine the internal structure of this model. They were. Convergent validity is supported if following criteria are achieved: the standardized factor loadings ≥ 0.71, individual item reliability ≥ 0.50, CR ≥ 0.60, and AVE ≥ 0.50 (Fornell and Larcker, 1981; Hair et al., 2009). In addition, discriminant validity is supported if 1 did not fall into the range of the bootstrapped 95% confidence intervals (CIs) of the inter-correlation coefficients among all latent variables (Torkzadeh et al., 2003).

After the overall and the internal structure of the theoretical model was verified, multiple-group CFAs (MG-CFAs) were implemented to examine the measurement invariance to verify that the scale was equally suitable for assessing the learning interest of both boys and girls. A series of five models was set to test measurement invariance with the latter models stringently constrained more parameters to be identical across two groups. The first model is a configural model. It verifies whether the number and patterns of factors are identical between models for boys and girls. The second model is the metric invariance model, and it was tested once the first model was demonstrated. The metric model tests whether the factor loading pattern in the models for the two groups was the same. The third model is the scalar model, and it was set to be tested once the second model was proved. The scalar model tests whether models for boys and girls have identical intercepts of items. The fourth model is the factor variances model, and it is tested based on scalar invariances. The factor variances model primarily investigates whether inter-correlations among factors and factor variances in the models for two groups were identical. The last and the most stringent is the residual variances model, and it is tested based on the invariances of factor variances. The residual variances model tests whether residual variances of items in the models for different groups were identical (Cheung and Rensvold, 2002; Wang and Wang, 2012). The configural model serves as the baseline model against the metric model. CFI and TFI ≥ 0.95, RMSEA ≤ 0.08, ΔCFI ≤ 0.01, and ΔTLI ≤ 0.02 are considered supportive evidence for the more stringent model (Hu and Bentler, 1999; Cheung and Rensvold, 2002).

## Results

### Measurement Models of Teacher Rating Scale of Learning Interest

Table 1 presents the goodness of fit and the indices for comparing parsimoniousness of all learning interest models. For the 1-factor model, all fit indices failed to meet the criteria for a well-fitted model: $\chi_{\left(20,\text{N}=132\right)}^{2}$ = 118.06 (*p* < 0.05), CFI = 0.891, TLI = 0.848, RMSEA = 0.193 (90% CI ranged from 0.160 to 0.227). In contrast, the 2-factor model fits data well: $\chi_{\left(19,\text{N}=132\right)}^{2}$ = 22.53 (*p* > 0.05), CFI = 0.996, TLI = 0.994, RMSEA = 0.038 (90% CI ranged from 0.000 to 0.088). Despite the 3-factor model also fits data well: $\chi_{\left(17,\text{N}=132\right)}^{2}$ = 20.82 (*p* > 0.05), CFI = 0.996, TLI = 0.993, RMSEA = 0.041 (90% CI ranged from 0.000 to 0.093). χ^2^-values for both 2- and 3-factor models were not significant, and the CFIs and TLIs of these two models were all above 0.95, and RMSEA values of which were below 0.08. It showed that the 3-factor model was not significantly better than the 2-factor model, and the 3-factor model was more complex and did not contribute to better model fit. In addition, the 2-factor model also showed the lower values on AIC and BIC comparing to the alternative models. This demonstrated that the 2-factor model was the best model for understanding kindergarteners' learning interest.

**Table 1:** Goodness of fit and comparisons of all models.

**Model**	**χ^2^**	***df***	**CFI**	**TLI**	**RMSEA [90%CI]**	**Δ*χ*^2^**	**AIC**	**BIC**
1-factor	118.06^*^	20	0.891	0.848	0.193 [0.160–0.227]	-	1951.29	2020.48
2-factor	22.53	19	0.996	0.994	0.038 [0.000–0.088]	95.53^*^	1804.27	1876.34
3-factor	20.82	17	0.996	0.993	0.041 [0.000–0.093]	1.71	1805.49	1883.33

* *p < 0.05*.

### Internal Quality of Measurements

After the best theoretical model was identified, the internal quality of measurement was further investigated on this basis. For the 2-factor model, standardized factor loadings for situational interest items were 94, 0.95, 0.91, and 0.93, respectively, and for individual interest items were 92, 0.94, 0.95, and 0.96, respectively. Individual item reliabilities for the former were 88, 0.90, 0.83, and 0.86, respectively, and for the latter were 85, 0.88, 0.90, and 0.92, respectively. It was clear that all the standardized factor loadings and individual item reliabilities were above 0.71 and 0.50, respectively. All the CRs and AVEs were above 0.60 (0.96 for situational interest and 0.97 for individual interest) and 0.50 (0.87 for situational interest and 0.89 for individual interest), respectively. The bootstrap 95% CIs of inter-correlations among latent variables ranged from 0.85 to 0.93 (1 did not fall into this range). It showed that both convergent validity and discriminant validity of the 2-factor learning interest model were supported.

### Measurement Invariance

Multiple-group CFAs were conducted to examine whether the 2-factor learning interest measurement is invariant across genders, and the results are presented in Table 2. As it can be seen, all CFIs and TLIs were greater than the cutoff value of 0.95 in all models, and the majority of RMSEAs were below 0.08 (except for the configural and the factor variances models, but 0.08 was included in the range of 90% CI of RMSEA). The ΔCFIs were ≤ 0.01 and ΔTLIs ≤ 0.02, as subtracted the corresponding values of the configural model from the metric model and subtracted which of the scalar model from the metric model.Similarly, factor invariances can be also considered achieved because the RMSEA and ΔCFI were only slightly greater than 0.80 (it fell into the range of 90% CI) and 0.01, but ΔTLI was below 0.02 when the factor invariances model was compared to the scalar model. Finally, invariance of residual variances was demonstrated due to the RMSEA being below 0.80, the ΔCFI was below 0.01, and ΔTLI was also below 0.02 when the corresponding values of the factor invariances model were subtracted from the residual variances model. Consequently, the residual variances model was demonstrated to be the final model representing factor patterns, factor loadings, intercepts of items, factor means, inter-correlations among latent factors, factor variances, and residual variances of items holds identical and the related coefficients could be compared across gender.

**Table 2:** Measurement invariance tests of the 6-factor model.

**Model**	**χ^2^**	***df***	**Δ*χ*^2^**	**RSEAM[90%CI]**	**CFI**	**ΔCFI**	**TLI**	**ΔTLI**
1. Configural	57.77	38	-	0.089 [0.036–0.133]	0.979	-	0.970	-
2. Metric	60.58	44	2.81	0.076 [0.005–0.119]	0.983	0.004	0.978	0.002
3. Scalar	70.38	52	9.8	0.073 [0.011–0.114]	0.981	0.002	0.979	0.001
4. Factor variances	86.24	55	15.86	0.093 [0.052–0.129]	0.968	0.013	0.967	0.012
5. Residual variances	87.29	63	1.05	0.076 [0.030–0.113]	0.975	0.008	0.978	0.011

## Discussion and Implications

The main purpose of this study was to develop and validate an instrument for measuring and identifying kindergarteners' learning interests. The results indicated that the 2-factor learning interest model, composed of situational interest and individual interest, maybe the most suitable model for understanding kindergarteners' learning interests. The convergent and discriminant validity of this scale was also good. Testing measurement invariance for boys and girls suggests the 2-factor model could be compared across genders.

The results indicated that kindergartens may have already developed situational interest and individual interest in language learning. However, these findings supported the learning interest theory developed in the early stage but not the one which developed later. In addition, the findings also provided some supportive empirical evidence suggesting kindergarteners' learning interest may be either triggered and maintained by instructional design or motivated by intrinsic enjoyment or curiosity (Harackiewicz et al., 2008; Linnenbrink-Garcia et al., 2010; Knogler et al., 2015).

For measurement invariances across genders based on the validated learning interest scale, it suggested that the teacher rating scale is a valid and an easy way for a teacher to evaluate the state and changes of kindergarteners' learning interest in the learning process and toward specific instructional elements (i.e., certain teaching method or material). However, it does not mean that kindergarteners are considered to have limited capability in self-reporting their learning interests. In contrast, the latest empirical evidence suggested that children as young as 5 years may be able to evaluate their achievement motivation (Wu, 2022). These findings suggested that the teacher rating scale of learning interest could be an efficient way to investigate kindergarteners' learning interests and the effect of teaching or instruction on kindergarteners' learning interests. In addition, the content of this scale could be also taken as a basis for constructing an appropriate instrument for kindergarteners to rate themselves.

Concerning the possible effect of the same pseudonyms used in each item on the teacher ratings for different groups, the results demonstrated that boy's pseudonyms do not affect teacher's ratings for girls because a strict measurement invariance is supported across genders. It shows that teachers can get the gist of the items and their ratings are not affected by the pseudonym used in each item. This finding suggested there may be no need for the researcher to design different versions or to use different pseudonyms, respectively, for rating boys and girls. In short, a teacher rating scale of learning interest is sufficient for a teacher to rate different gender groups of kindergarteners. It may serve as useful information complementary to a recent study that used different measurement versions for different gender groups. Finally, the teacher rating scale of learning interest was developed specifically to measure learning interest for language; the results demonstrated the dimensionality of learning interest for language is similar to studies measuring learning interest for science classes in high school and psychology in the introductory psychology class in the University (Linnenbrink-Garcia et al., 2010; Knogler et al., 2015). It may suggest that there is a generality of the 2-dimensional structure of learning interest.

## Limitations and Future Directions

The limitations of this study primarily focused on three aspects: the representativeness of the sample, dimensionality and field specificity of learning interest, and the time point of implementing the measurement. First, as the sample was selected by a nonrandom stratified method and this study served as a preliminary study of kindergarteners' learning interest, the construct validity or dimensionality of the teacher rating scale of learning interest based on a 2-factor model for interpreting kindergarteners' learning interest may not necessarily be appropriate for other samples with the same or below this age. In addition, although recent empirical evidence suggesting kindergarteners' achievement motivation may be effectively investigated by their teachers, but to what extent teacher rating could reflect the authenticity of kindergarteners' learning interest remains unclear. Further research is needed to contribute to the discussion on this topic. Second, although the dimensionality measurement invariances of learning interest are demonstrated on kindergarten boys and girls aged 5 years, these results are based on teacher ratings. It remains unclear to what extent these results are consistent with self-reporting by kindergarteners themselves. Future studies can develop a scale that is suitable for kindergarteners to rate their learning interest by themselves, and examine its dimensionality. In addition, it is also encouraged that researchers develop two versions with equivalent meaning or gist in each item, respectively, for measuring kindergarten boys' and girls' learning interests, and to test the dimensionality of the learning interest and the measurement invariances between these two versions. About the field specificity and generality of learning interest, results of the dimensionality of learning interest were drawn based on the teacher's observations in the language class. However, a similar factorial structure is also demonstrated in a different class at different education levels. More studies are needed to further clarify the field specificity and generality of learning interests at the kindergarten level. Lastly, although the 4-factor model of learning interest was not supported by the present findings, it may be because interest needs some time to develop and/or to be discovered according to the four-stage learning interest theory. It is unknown whether some or most kindergarteners are still under development while teachers are rating due to large individual differences. More studies implemented at different time points may be beneficial for clarifying this consideration.

## Data Availability Statement

The original contributions presented in the study are included in the article, further inquiries can be directed to the corresponding author.

## Ethics Statement

Ethical review and approval was not required for the study on human participants in accordance with the local legislation and institutional requirements. Written informed consent for participation was not required for this study in accordance with the national legislation and the institutional requirements.

## Author Contributions

The author confirms being the sole contributor of this work and has approved it for publication.

## Funding

This study was supported by the Taiwan Hakka Affair Council (grant no. 110HB01731).

## Conflict of Interest

The author declares that the research was conducted in the absence of any commercial or financial relationships that could be construed as a potential conflict of interest.

## Publisher's Note

All claims expressed in this article are solely those of the authors and do not necessarily represent those of their affiliated organizations, or those of the publisher, the editors and the reviewers. Any product that may be evaluated in this article, or claim that may be made by its manufacturer, is not guaranteed or endorsed by the publisher.
